# First Whole-Genome Sequence of *Mycobacterium iranicum*, a Newly Reported Mycobacterial Species

**DOI:** 10.1128/genomeA.00732-13

**Published:** 2013-09-26

**Authors:** Joon Liang Tan, Hien Fuh Ng, Wei Yee Wee, Mia Yang Ang, Guat Jah Wong, Yun Fong Ngeow, Siew Woh Choo

**Affiliations:** Department of Medical Microbiology, Faculty of Medicine, University of Malaya, Kuala Lumpur, Malaysiaa; Dental Research and Training Unit, Faculty of Dentistry, University of Malaya, Kuala Lumpur, Malaysiab; Genome Informatics Research Laboratory, High Impact Research (HIR) Building, University of Malaya, Kuala Lumpur, Malaysiac

## Abstract

*Mycobacterium iranicum* is a new species of nontuberculous mycobacterium reported in 2013. Here, we describe the first whole-genome sequence of this species, that of *M. iranicum* strain UM_TJL, isolated from a patient in Malaysia.

## GENOME ANNOUNCEMENT

*Mycobacterium iranicum* is a novel, rapidly growing, and scotochromogenic mycobacterial species first named in 2013 by Shojaei et al. (1), who described eight strains from 6 countries on three different continents. The species was named after the country Iran, in which attention was first focused on this novel mycobacterium. A pathogenic role for *M. iranicum* has been suggested in some patients with pulmonary infections, such as pneumonia, chronic obstructive airway disease, and bronchiectasis (1, 2). However, the clinical significance of this mycobacterium is still unclear. Phylogenetic analysis based on a few housekeeping genes showed that *M. iranicum* is closely related to another rapid grower, *Mycobacterium gilvum* (1). Further investigations on the genomics, biology, epidemiology, and pathogenicity of this mycobacterium will be needed to clarify its status as an emerging human pathogen.

The draft genome sequence of *M. iranicum* strain UM_TJL was shotgun sequenced using the Illumina HiSeq 2000 paired-end sequencing platform. The 36,709,303 reads obtained were preprocessed and *de novo* assembled with the CLC Genomics Workbench 5.1, resulting in 119 contigs, with an N_50_ contig size of 116,366 bp (range, 243 bp to 334,562 bp). The size of this sequenced genome is 6,137,283 bp, with a G+C content of 66.1%.

Annotation of the sequenced genome was performed using the Rapid Annotations using Subsystems Technology (RAST) pipeline (3). RAST predicted 6,045 putative open reading frames (ORFs), including 5,995 predicted coding sequences (CDSs) and 50 RNAs (47 tRNAs and 3 rRNAs). RAST functional analysis of the predicted protein-coding genes showed 78 genes involved in the synthesis of the cell wall and capsule, 89 in membrane transport, 227 in protein metabolism, 141 in DNA metabolism, 149 in respiration, and 522 in amino acid and derivative metabolism. No genes were predicted in the photosynthesis process, motility, and chemotaxis categories.

A genomic island (GI) is a part of the genome that contains a cluster of genes that were most likely acquired by horizontal gene transfer. GIs are often responsible for microbial adaptations and therefore have a significant impact on bacterial evolution (4). We predicted GIs in the *M. iranicum* strain UM_TJL using the IslandViewer software (5). This analysis revealed 13 putative GIs that might contribute to the diversification, evolution, drug resistance, and probably virulence of this pathogen. One GI carries 2 putative genes encoding a small multidrug resistance transporter protein, EmrE, which might confer multidrug resistance in this human pathogen.

In conclusion, we report the genome sequence of UM_TJL, which to the best of our knowledge is the first genome sequence of the newly discovered species *M. iranicum.*

### Nucleotide sequence accession number.

The draft genome sequence of *M. iranicum* UM_TJL has been deposited in NCBI GenBank under the accession no. AUWT00000000.
